# Photocontrolled reversible self-assembly of dodecamer nitrilase

**DOI:** 10.1186/s40643-017-0167-3

**Published:** 2017-08-04

**Authors:** Qiao Yu, Yong Wang, Shengyun Zhao, Yuhong Ren

**Affiliations:** 1 East China University of Science and Technology, Shanghai, China; 2 Key Laboratory of Synthetic Biology, Institute of Plant Physiology and Ecology, Shanghai Institutes for Biological Sciences, Chinese Academy of Sciences, Shanghai, China; 3 Fujian Key Laboratory of Eco-Industrial Green Technology, Wuyi University, Wuyishan, China; 4 State Key Laboratory of Bioreactor Engineering, New World Institute of Biotechnology, East China University of Science and Technology, Shanghai, China

**Keywords:** Disassembly, iLID, Nitrilase, Photoswitch, Self-assembly

## Abstract

**Background:**

Naturally photoswitchable proteins act as a powerful tool for the spatial and temporal control of biological processes by inducing the formation of a photodimerizer. In this study, a method for the precise and reversible inducible self-assembly of dodecamer nitrilase in vivo (in *Escherichia coli*) and in vitro (in a cell-free solution) was developed by means of the photoswitch-improved light-inducible dimer (iLID) system which could induce protein–protein dimerization.

**Results:**

Nitrilase was fused with the photoswitch protein AsLOV2-SsrA to achieve the photocontrolled self-assembly of dodecamer nitrilase. The fusion protein self-assembled into a supramolecular assembly when illuminated at 470 nm. Scanning electron microscopy showed that the assembly formed a circular sheet structure. Self-assembly was also induced by light in *E. coli*. Dynamic light scattering and turbidity assay experiments showed that the assemblies formed within a few seconds under 470-nm light and completely disassembled within 5 min in the dark. Assembly and disassembly could be maintained for at least five cycles. Both in vitro and in vivo, the assemblies retained 90% of the initial activity of nitrilase and could be reused at least four times in vitro with 90% activity.

**Conclusions:**

An efficient method was developed for the photocontrolled assembly and disassembly of dodecamer nitrilase and for scaffold-free reversible self-assembly of multiple oligomeric enzymes in vivo and in vitro, providing new ideas and methods for immobilization of enzyme without carrier.

**Electronic supplementary material:**

The online version of this article (doi:10.1186/s40643-017-0167-3) contains supplementary material, which is available to authorized users.

## Background

Protein assembly has been extensively studied (King and Lai 2013; Yu et al. 2015), and represents a useful tool for biology and bioengineering processes owing to its potential applications in protein material development nanobiotechnology, and multienzyme biocatalysis (Matsumoto et al. 2016; Smith et al. 2011; Fairman and Åkerfeldt 2005; Holmes 2002). Although various protein assembly strategies have been reported, such as coimmobilization, protein fusion, protein crosslinking, and scaffold-mediated multienzyme colocalization (King and Lai 2013; Schoffelen and van Hest 2012; Brady and Jordaan 2009), those strategies do not have the ability to achieve the precise control of the assembly process, or realize the reversible disassembly in cell-free solutions (in vitro) or in bacterial cell cultures (in vivo). Including a control mechanism would solve this problem. Optogenetics provides a powerful tool for controlling biological processes through several light receptors such as cryptochromes (Kennedy et al. 2010), phytochrome B, phototropins (LOV), and rhodopsins (Levskaya et al. 2009; Yin and Wu 2013). Thus, we aimed to use light for the highly tunable, nontoxic, simple, and high-resolution spatiotemporal control of protein assembly. We focused on using the photosensitive LOV2 domain from *Avena sativa* (oat) (AsLOV2) (Strickland et al. 2010). AsLOV2 consists of a core Per-Arnt-Sim (PAS) domain that is sensitive to blue light and a C-terminal helical extension (Jα). Upon irradiation with 470-nm blue light, a covalent adduct is generated between a conserved cysteine residue in the Per-Arnt-Sim core of AsLOV2 and flavin mononucleotide (FMN) C(4a), leading to the unfolding of the Jα helix (Halavaty and Moffat 2007; Harper et al. 2004; Guntas et al. 2014). Several strategies have been developed to utilize AsLOV2 as a photoswitch, including using tunable light-inducible dimerization tags to control the interaction between the AsLOV2 domain and an engineered PDZ domain, and using the improved light-inducible dimer (iLID) system to control the location of transmembrane proteins (Guntas et al. 2014). The iLID system consists of an AsLOV2 mutant domain fused to the N-terminal of SsrA (called iLID-micro) and a 13-kDa adaptor protein, SspB, that can dimerize spontaneously and bind to SsrA (Guntas et al. 2014). After irradiation with 470-nm blue light, the conformation of the AsLOV2 domain changes, leading to the exposure of SsrA, which subsequently binds to SspB (Guntas et al. 2014; Zimmerman et al. 2016). The iLID presents several advantages, such as possessing strictly monomeric components, being easily expressed in *Escherichia coli*, and having a broad dynamic range, a highly tunable affinity, and a fast reversion rate in the dark (Guntas et al. 2014).

In this study, the dodecamer nitrilase from *Burkholderia cenocepacia* J2315 (Wang et al. 2013) (BCNIT) was selected as the model enzyme, and a light-controllable enzyme assembly method was developed using iLID and BCNIT. Dynamic light scattering (DLS), fluorescence complementation, scanning electron microscopy (SEM), optical density measurements (OD_600_), and enzyme activity assays were performed to study the mechanisms involved.

## Methods

### Materials

PrimeSTAR^®^ Max DNA polymerase (Takara Biotech, Dalian, China) was used for PCR amplification. The sequence information for AsLOV2-SsrA and SspB was acquired from the Protein Data Bank (4WF0 and 1YFN) and the genes and primers were synthesized (Generay, Shanghai, China) with codon optimized for expression in *E. coli*. FMN, mandelonitrile, and mandelic acid were purchased from Sigma-Aldrich (Shanghai, China). All other chemicals were purchased from SinopHarm Chemical Reagent Co., Ltd. (Shanghai, China). The BCNIT gene was acquired from our laboratory.

### Construction of fusion genes

The plasmids pET28a/BCNIT-AsLOV2-SsrA and pET28a/SspB were constructed by PCR cloning using PrimeSTAR^®^ Max DNA polymerase. The oligonucleotide sequence of the AsLOV2-SsrA domain containing 5′ *Spe*I and 3′ *Xho*I restriction sites was constructed on pET28a, and the flexibility linker of (GGGGS)_2_ was modified on the N terminal of AsLOV2-SsrA using the primers P3/R3. The BCNIT gene with 5′ *Nde*I and 3′ *Spe*I restriction sites was amplified by PCR based on the BCNIT template using the primers P2/R2. The genes were sequentially inserted into a modified pET28a plasmid with *Spe*I restriction sites, producing the pET28a/BCNIT-AsLOV2-SsrA (BNAS) domain, and then pET21a/BCNIT-AsLOV2-SsrA was constructed by inserting the BNAS domain into pET21a. The SspB gene was prepared by gene synthesis and constructed on pET21a and a pET21c plasmid modified with a resistance gene against chloromycetin instead of ampicillin. The sequences mN159 and mC160 were inserted into pET21a/BCNIT-AsLOV2-SsrA and pET28a/BCNIT-AsLOV2-SsrA using the primers P4/R4 and P5/R5 to produce the plasmids pET21a/BCNIT-mN159-AsLOV2-SsrA (BNMnAS) and pET28a/BCNIT-mC160-AsLOV2-SsrA (BNMcAS), respectively. The plasmids were transfected into *E. coli* BL21 (DE3) for recombinant protein expression. All the primers used are shown in Table 1. The detail information of construction of fusion genes is described in the supporting information (See Additional file 1).

**Table 1:** Primers used for cloning and transcript amplification in this study

Primer	Sequence (5′–3′)
P1	GGAATTCCATATGGAATACAAATCCTC
R1	CCGCTCGAGTTATTCATCGTAGATTTCTTCAG
P2	GGAATTCCATATGACCATCAATCACCCG
R2	GACTAGTCGAGCCACCGCCACCAGCGGGTGTGACGCGC
P3	GGACTAGTGGCGGTGGCGGATCTTTAGCCACTACTTTAGAAAGG
R3	ATAAGAATGCGGCCGCAAAATAATTTTCATCATTAG
P4	AGGTGGCTCTACTAGTGGCGGAGGTGGCTCTGTG
R4	CACCTCCGCCACTAGTCGATCCGCCACCGCCGTC
P5	AGGTGGCTCTACTAGTGGCGGTGGCGGATCTGGC
R5	CACCTCCGCCACTAGTAGAGCCACCTCCGCCGCT

### Protein expression and purification

Recombinant *E. coli* BL21 (DE3) cells were cultured in Luria–Bertani medium at 37 °C until reaching an optical density at 600 nm (OD_600_) of 0.5–0.7. Protein expression was induced by adding 0.1 mM isopropyl β-d-1-thiogalactopyranoside and incubating the cells at 18 °C for 20 h. The BCNIT, BNAS, and SspB proteins were purified with Ni-nitrilotriacetic acid columns (GE Healthcare, Waukesha, WI, USA). Protein concentrations were determined using the Bradford assay (Beyotime Biotechnology, Shanghai, China).

### Optical control of enzyme assembly and disassembly in *E. coli*

The assembly of the fusion proteins in *E. coli* was monitored using the fluorescence complementation assay (Gao et al. 2015). The three plasmids pET21a/BNMnAS, pET28a/BNMcAS, and pET21c/SspB were cotransfected into *E. coli* BL21 (DE3). After centrifugation at 8000×*g* for 10 min and resuspension of the cell pellets in 5 mL 20 mM phosphate-buffered saline (PBS; pH 7.4), 10 µM FMN was added and the cells were incubated for 10 min at 37 °C under a 470-nm blue light-emitting diode array (Guntas et al. 2014). Meanwhile, an equivalent number of cells coexpressing BNMnAS, BNMcAS, and SspB in the dark, and cells coexpressing mN159, mC160, and SspB under 470-nm light were used as controls and assayed in the same manner. We monitored the differences between the samples placed under the blue light and those kept in the dark by measuring epifluorescence (excitation: 562–640 nm, emission: 590–650 nm).

### Optical control of enzyme assembly and disassembly in vitro

The BNAS fusion proteins were assembled on SspB by triggering the affinity between AsLOV2-SsrA and SspB under illumination at 470 nm. Self-assembly via photopolymerization occurred in PBS buffer (pH 7.4) after mixing the freshly purified BNAS and SspB proteins at an equimolar ratio under freezing conditions and adding 10 µM FMN to induce a stable supramolecular polymerization. The disassembly measurement was performed in the dark after 470-nm blue light illumination. The optical density at 600 nm (OD_600_) was used to characterize the turbidity of the supramolecular assembly (Kanekura et al. 2016). The reactions before measured the OD_600_ were carried out in an ice bath. Moreover, the assemblies were visualized using scanning electron microscopy. BNAS or BCNIT and SspB were mixed at a 1:1 molar ratio.

### Dynamic light scattering (DLS) assay

To explore the assembly and disassembly before and after illumination, we used a DynaPro NanoStar^®^ instrument (Wyatt Technology, Santa Barbara, CA, USA) to analyze the changes in the particle size of the BNAS–SspB (BNASS) supramolecular complex. Samples were filtered with a pore size of 0.22 μm prior to analysis, and each measurement was repeated three times at 18 °C.

### Field-emission scanning electron (FESEM)

The sample was repeatedly washed with deionized water and resuspended, dried under air, and applied onto a slide. Images were collected on an S4800 scanning electron microscope (Hitachi, Tokyo, Japan) operated at 15 kV. In order to more clearly observe morphology and structure of BNASS supramolecular assemblies, we collected images with different resolutions.

### Enzymatic activity assay in vitro and in vivo

Nitrilase activity was measured using reverse-phase high-performance liquid chromatography by monitoring the decrease of mandelonitrile (substrate) or the increase of mandelic acid (product) at 210 nm. The standard assay mixtures contained 20 mM mandelonitrile, 10 µM FMN, 100 mM PBS (pH 7.4), and 6 µM pure enzyme (in vitro) or 10 mg/mL of *E. coli* cells expressing nitrilase (in vivo). After reaction at 30 °C with agitation at 200 rpm for 20 min, 100 μL of 1 M HCl was added to stop the reaction, and centrifugation was performed at 13,000×*g* for 10 min, before removing 500 μL of sample for reverse-phase high-performance liquid chromatography analysis using a Zorbax^®^ SB-Aq column (250 × 4.6 mm, 5 µm; Agilent Technologies, USA) at a detection wavelength of 210 nm (Ni et al. 2013). The detail information of enzymatic activity assay is described in the supporting information (See Additional file 1).

## Results and discussion

**Fig. 1:**
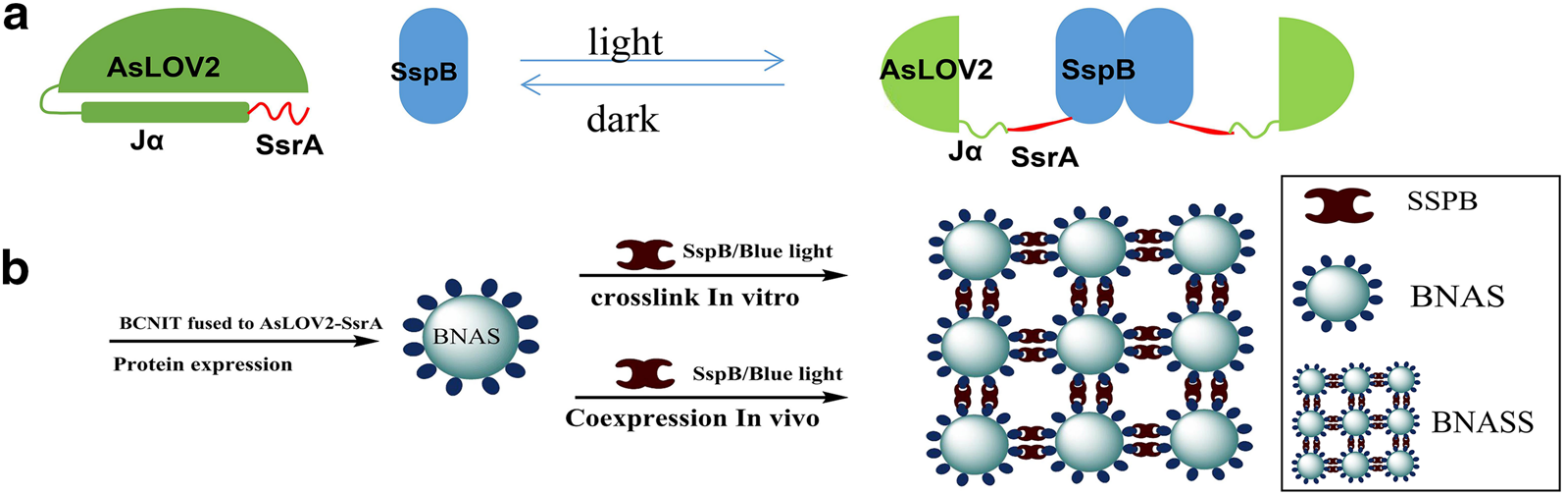
Schematic design of assembly and disassembly. **a** Schematic design of the improved light-inducible dimer system. **b** Strategy for the light-controlled dodecamer assembly and disassembly of nitrilase from *Burkholderia cenocepacia* J2315 (BCNIT) in cell-free solution (in vitro) and in *Escherichia coli* (in vivo)

The strategy for the light-controlled self-assembly of dodecamer nitrilase in vitro and in vivo is outlined in Fig. 1. The fusion protein BNAS could be assembled under 470-nm light, and the resulting assemblies could be disassembled in the dark.

### Light-controlled assembly and disassembly in vitro

**Fig. 2:**
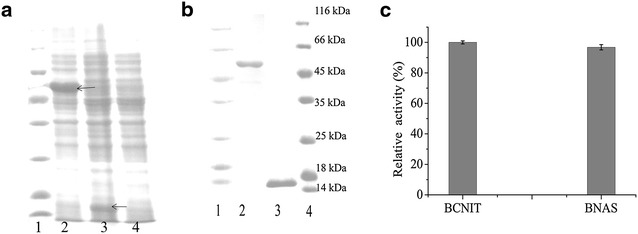
Expressions of BNAS and SspB proteins in *E. coli* BL21 (DE3) cells. **a** SDS-PAGE image of the cells expressing BNAS and SspB at 20 h after IPTG addition. *Lanes 1* marker; *lane 2* BNAS; *lane 3* SspB; *lane 4* control. The *arrow* shows the destination band. **b** SDS-PAGE analyses of purified BNAS and SspB in vitro. *Lanes 1* and *4* marker; *lane 2* purified BNAS; *lane 3* purified SspB. **c** Comparison of the enzyme activities of BCNIT and BNAS in vitro. The determination of nitrilase activity was performed at 30 °C for 30 min with 10 µM FMN under 470-nm blue light illumination. Under the experimental conditions, the relative activity was expressed as a percentage of the maximum activity. *Error bars* represent the standard error of three replicates

**Fig. 3:**
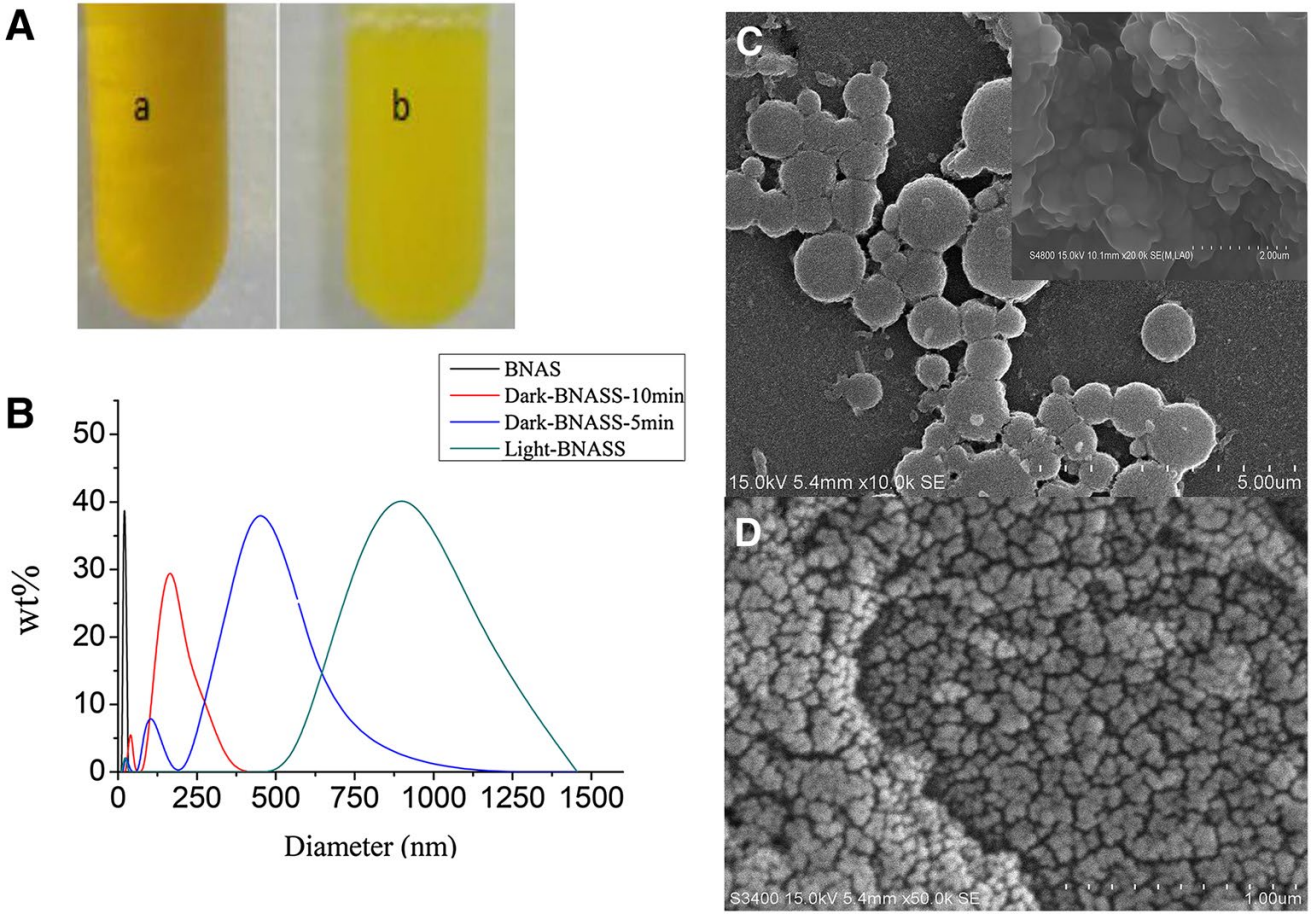
The light-controlled assembly, disassembly, and morphology of the BCNIT-AsLOV2-SsrA-SspB (BNASS) complex in vitro. **A** The solution states of the mixture of BNAS, SspB, and FMN either (*a*) kept in the dark or (*b*) exposed to light. **B** Dynamic light scattering analysis of the hydrodynamic diameter of pure BCNIT-AsLOV2-SsrA (BNAS) and the mixture of SspB and BNAS. **C** Structure of the BNASS complex in vitro visualized by FESEM and SEM after illumination with blue light. **D** Structure of the BNASS complex in vitro visualized by SEM when the light is removed for 10 min. The reactions were carried out in an ice bath

The in vitro light-controlled assembly was performed by expressing and purifying SspB and BNAS in *E. coli* BL21 (DE3). As shown in the Fig. 2a, the purified proteins were observed with the molecular mass of 13 and 58 kDa for SspB and BNAS, respectively. BNAS retained about 97% of the specific activity of BCNIT (Fig. 2b), indicating that the protein fused with AsLOV2-SsrA did not suffer any drastic structural change in vitro. The light-controlled assembly was triggered by mixing the purified SspB and BNAS proteins with FMN under illumination at 470 nm. Following illumination, the solution changed rapidly from clear to turbid (Fig. 3A), and the DLS measurements indicated that BNAS was converted into a BNASS supramolecular polymer (Fig. 3B). The hydrodynamic diameter of pure BNAS is about 30 nm. The hydrodynamic diameter of the proteins decreased from 1000 to 100 nm after the light was removed, suggesting that the supramolecular polymer was disassembled within 5 min. However, the assembly could not disassembled completely, which might result from a remnant weak affinity between SsrA and SspB in the dark.

**Fig. 4:**
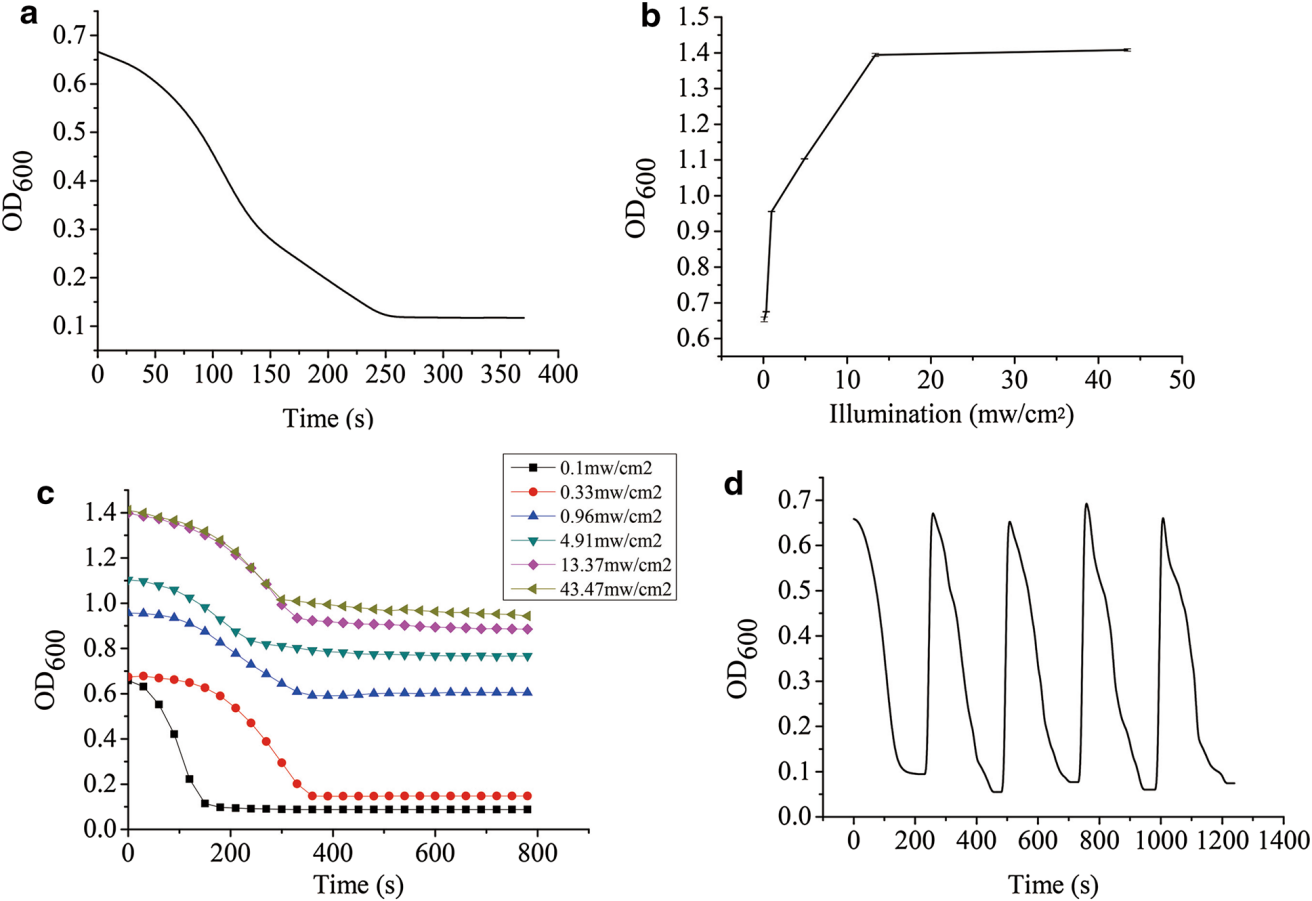
Characterization of the light-controlled reversible assembly and disassembly of the BNAS in vitro by measuring the optical density at 600 nm and the nitrilase activity of BNASS. **a** Changes in OD_600_ values with the disassembly of the BNASS complex. **b** Changes in OD_600_ values under different blue light intensities during photopolymerization. **c** Different degrees of disassembly of the polymer incubated under dark conditions after illumination at different intensities. **d** Light-induced reversible assembly and disassembly of the BNASS complex. The reactions were carried out in an ice bath. *Error bars* represent the standard error of three replicates

The morphology and structure of BNASS supramolecular polymers were observed by scanning electron microscopy (SEM), and it was found that the assembly formed a circular sheet structure with a hydrodynamic diameter about 1 μm (Fig. 3C). When the blue light was removed for 10 min, the hydrodynamic diameter of the assembly was found to vary from 30 to 200 nm by SEM (Fig. 3D). The results confirmed that SspB and AsLOV2-SsrA could mediate the formation of a supramolecular polymer under illumination at 470 nm and the polymer would be disassembled in the dark. Figure 4a shows the results obtained when monitoring the disassembly of the supramolecular polymer in the dark. The change of OD_600_ of the supramolecular polymer suggested that the supramolecular polymer disassembled dramatically, confirming the DLS results. An experiment was performed to examine whether different illumination intensities could influence the assembly under blue light and the disassembly in the dark. Figure 4b shows that the OD_600_ increased gradually with the increasing illumination intensities. This result indicated that the light intensity has a great effect on the enzyme assembly, possibly because the light intensity affected the responding time of the AsLOV2-SsrA to the blue light, resulting in different degrees of assembly at the same time. However, when the light was removed, assemblies with different OD_600_ values showed varying degrees of disassembly (Fig. 4c). This observation could be explained by larger assemblies exhibiting a stronger clustering effect (Pieters 2009; Sengupta et al. 2011; Jaenicke 2000), thereby weakening the degree of disassembly. The disassembly efficiency is optimal when the OD_600_ is 0.7–0.8. Therefore, we employed the assembly with an OD_600_ value of 0.7 in the following experiments.

The recyclability of multienzyme polymers is an important factor when it comes to applications in synthetic biology and biocatalysis, especially for light-controlled enzyme assembly processes. The recyclability of the light-controlled assembly was evaluated by examining the change in the OD_600_ value of the mixture of BNAS and SspB when the mixture solution was repeatedly transferred between blue light illumination and the dark conditions. As shown in Fig. 4d, the OD_600_ value decreased from 0.7 to 0.1 within 5 min in the dark, then increased to 0.7 within a few seconds under illumination at 470 nm, which is in accordance with the results displayed in Fig. 3B. Furthermore, neither the assembly nor the disassembly was affected by repeating the light/dark cycling up to five times.

**Fig. 5:**
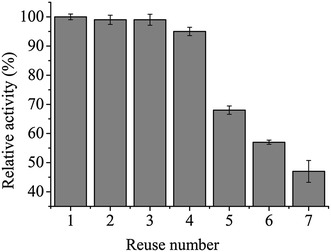
The relative nitrilase activity of BNASS after multiple assembly–disassembly cycles was expressed as a percentage of the maximum activity. The determination of nitrilase activity was performed at 30 °C for 30 min with 10 µM FMN under 470-nm blue light illumination. *Error bars* represent the standard error of three replicates

To further determine whether the catalytic activity of the assemblies is affected by repeated assembly–disassembly cycles, we analyzed the activity of the supramolecular assembly through seven cycles of illumination at 470 nm followed by incubation in the dark. Figure 5 shows that the light-controlled assembly retained a high level of activity as the number of cycles increased, and still retained 90% of its initial activity after four cycles. The dramatic decrease of enzyme activity after five or more cycles may be caused by the disruption of the enzyme assembles after the violent resuspension, the loss of some smaller enzyme assembles during centrifugal isolation, and the toxicity of nitrile substrate after multicycles.

### Light-controlled assembly in vivo

**Fig. 6:**
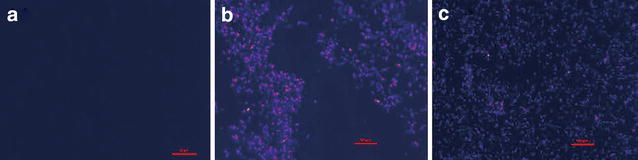
Fluorescence microscopy visualization of *Escherichia coli* BL21 cells coexpressing **a** mN159, mC160, and SspB under blue light, **b** BNMnAS, BNMcAS, and SspB under blue light. **c** BNMnAS, BNMcAS, and SspB in the dark. *Scale bar* 10 μm

**Fig. 7:**
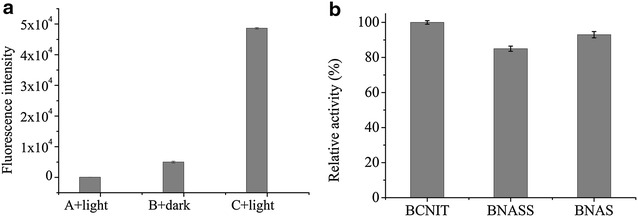
The fluorescence intensities of fusion protein-coexpressing strains. **a** A + light represents the strains coexpressing mN159, mC160, and SspB under blue light illumination; B + dark represents the strains coexpressing BCNIT-mN159-AsLOV2-SsrA (BNMnAS), BCNIT-mC160-AsLOV2-SsrA (BNMcAS), and SspB in the dark; C + light represents the strains coexpressing BNMnAS, BNMcAS, and SspB under blue light illumination. **b** Comparison of the nitrilase activities of BCNIT, BNAS, and BNASS in *E. coli*. *Error bars* represent the standard error of three replicates. The determination of nitrilase activity was performed at 30 °C for 30 min with 10 µM FMN under 470-nm blue light illumination. Under the experimental conditions, the relative activity was expressed as a percentage of the maximum activity. *Error bars* represent the standard error of three replicates

To assess whether the light-controlled assembly and disassembly of the fusion proteins can occur within *E. coli* cells, BNAS and SspB were coexpressed in *E. coli* BL21 (DE3), and both of them could be expressed in their soluble forms. To monitor the light-controlled formation of the assemblies in vivo, a fluorescence complementation assay was also performed. The red fluorescent protein mCheery was divided into two parts, mN159 and mC160, and was fused to the site between BCNIT and AsLOV2-SsrA, thus producing the BCNIT-mN159-AsLOV2 (BNMnAS) and BCNIT-mC160-AsLOV2 (BNMcAS), respectively. Little fluorescence was observed when mN159, mC160, and SspB were coexpressed in *E. coli* BL21 (DE3) and the cells were illuminated under blue light (Fig. 6a). However, the fluorescence intensity increased markedly when BNMnAS and BNMcAS were coexpressed with SspB under illumination at 470 nm (Fig. 6b), indicating that the strong fluorescence is due to the presence of AsLOV2-SsrA. Concordant with the increase of fluorescence intensity resulting from induction by blue light illumination, the strains coexpressing BNMnAS, BNMcAS, and SspB showed significantly lower fluorescence intensities in the dark (Fig. 6c). Although the core domain of the AsLOV2 covered the binding site of the interaction between SsrA and SspB in darkness, the SsrA domain was not completely inactivated, and hence SsrA and SspB kept a weaker affinity in darkness (Guntas et al. 2014). The weaker affinity led to the assembly of fewer nitrilases, and thus there were weak fluorescent signals in the dark. At the same time, the fluorescence intensity of 5.0 OD_600_ cells collected after induction was measured using a Spectra Max M5 microplate reader (Synergy Mx, Bio-Tek Instruments, Inc., Winooski, VT, USA). As shown in Fig. 7a, the results of fluorescence intensities are exactly the same as those shown in Fig. 6. All results showed that the strong fluorescence signal is due to the presence of AsLOV2-SsrA and blue light. To determine the catalytic activity of BNASS in vivo, BNAS and SspB were coexpressed in *E. coli* BL21. As a control, the same number of cells expressing BNAS and BCNIT were analyzed following the same procedure. Similar to the result obtained in vitro, as shown in Fig. 7b, the activity of BNAS in vivo remained at 93% relative to that of BCNIT. The supramolecular assembly BNASS also maintained approximately 85% activity relative to that of BCNIT regardless of light or dark exposure. The results show that the supramolecular assembly process barely changed the activity of BCNIT.

## Conclusions

In conclusion, a strategy for the light-controlled assembly and disassembly of oligomeric enzymes through an optogenetic tool was successfully applied to in vitro and in vivo systems. The light-controlled assembly did not affect the catalytic ability of enzymes, and the assemblies showed good reusability and reversibility. The results indicated that the construction of supramolecular assemblies mediated by AsLOV2 can provide a solid biocatalyst with good reusability and reversibility in cell-free solutions and bacterial cultures, and this method might provide a promising way to precisely and reversibly control protein–protein interactions in mammalian cells. These results indicate that the photocontrolled self-assembly strategy is a powerful tool for achieving the scaffold-free self-assembly and immobilization without carrier of enzymes. Thus, the photocontrolled enzyme assembly strategy may be used in the multienzyme biocatalysis to realize precise and reversible control of the process of metabolic enzyme cascades in vivo and in vitro.

## Additional file

**Additional file 1.** Supporting Information.

## Abbreviations

AsLOV2: LOV2 domain isolated from *Avena sativa*
BCNIT: nitrilase from *Burkholderia cenocepacia* J2315
BNAS: BCNIT-AsLOV2-SsrA domain
BNASS: BNAS-SspB
BNMnAS: BCNIT-mN159-AsLOV2-SsrA
BNMcAS: BCNIT-mC160-AsLOV2-SsrA
CRY: cryptochromes
DLS: dynamic light scattering
FMN: flavin mononucleotide
HPLC: high-performance liquid chromatography
iLID: photoswitch-improved light-inducible dimer
IPTG: isopropyl β-d-1-thiogalactopyranoside
LB: Luria broth
LOV: light-oxygen-voltage
OD: optical density
PAS: Per-Arnt-Sim
PhyB: phytochrome B
FESEM: field-scanning electron microscopy
