# Supplementary material for: Photocontrolled reversible self-assembly of dodecamer nitrilase
Source: Bioresour Bioprocess. 2017 Aug 4;4(1):36. doi: 10.1186/s40643-017-0167-3 (PMC5544783; doi:10.1186/s40643-017-0167-3)
Supplement: Supplementary file 1 — Additional file 1. Supporting Information. [file 40643_2017_167_MOESM1_ESM.docx]

**Fluorescence complementation assay**

### The SspB gene with *Nde*I and *Xho*I was constructed on the PET21a using the primers P1 (GGAATTCCATATGGAATACAAATCCTC) /P2 (CCGCTCGAGTTATTCATCG TAGATTTCTTCAG). The BCNIT gene with 5′ *Nde*I and 3′ *Spe*I restriction sites was amplified by PCR based on the BCNIT template using the primers P2 (GGAATTCCATATGACCATCAATCACCCG)/ R2 (GACTAGTCGAGCCACCGCC ACCAGCGGGTGTGACGCGC) Oligonucleotide sequences of the AsLOV2-SsrA domain with 5′ *Spe*I and 3′ *Xho*I restriction sites was constructed on the pET28a, and the flexibility linker of (GGGGS)×2 was modified on the N terminal of the AsLOV2-SsrA using the primers P3 (GGACTAGTGGCGGTGGCGGATCTTTAGC CACTACTTTAGAAAGG) /R3 (ATAAGAATGCGGCCGCAAAATAATTTTCATC ATTAG). The genes were sequentially inserted into a modified pET28a plasmid with *Spe*I restriction sites, producing the pET28a-BCNIT-AsLOV2-SsrA domain (BNAS), and then constructed the pET21a-BCNIT-AsLOV2-SsrA by inserted the BCNIT-AsLOV2-SsrA into the pET21a. The SspB gene modified pET21c, which transform its resistance of ampicillin into [chloromycetin](D:/Dict/6.3.69.8341/resultui/frame/javascript:void(0);). The sequences of mM159 and mC160 were inserted into pET21a-BCNIT-AsLOV2-SsrA and pET28a-BCNIT-AsLOV2-SsrA using the primers of P4 (AGGTGGCTCTACTAGTG GCGGAGGTGGCTCTGTG)/R4 (CACCTCCGCCACTAGTCGATCCGCCACCGC CGTC) and P5 (AGGTGGCTCTACTAGTGGCGGTGGCGGATCTGG C)/R5 (CACCTCCGCCACTAGTAGAGCCACCTCCGCCGCT) respectively, and producing the plasmids of pET21a-BCNIT-mN159-AsLOV2-SsrA (BNMnAS); pET28a-BCNIT-mC160-AsLOV2-SsrA (BNMcAS). The plasmids were transfected into *E. coli* BL21 (DE3) for recombinant protein expression. All primers are shown in the table1.

**Enzymatic activity assay**

Nitrilase activity was measured using reverse-phase high-performance liquid chromatography by monitoring the decrease of [mandelonitrile](D:/Dict/6.3.69.8341/resultui/frame/javascript:void(0);) (substrate) or the increase of [mandelic](D:/Dict/6.3.69.8341/resultui/frame/javascript:void(0);) [acid](D:/Dict/6.3.69.8341/resultui/frame/javascript:void(0);) (product) at 210 nm.

*In vitro*, the standard assay mixtures contained 20 mM [mandelonitrile](D:/Dict/6.3.69.8341/resultui/frame/javascript:void(0);), 10 µM FMN, 100 mM PBS (pH 7.4), and 6 µM pure enzyme. When assess the activity of BNASS, BNAS and SspB were mixed at a molar ratio of 1: 1 under blue light and reacted for 5 min, followed by centrifugation to obtain an assembly precipitate, resuspension. After reaction at 30 °C with agitation at 200 rpm for 20 min under the blue light and in the dark respectively, 100 µL of 1 M HCl was added to stop the reaction, and centrifugation was performed at 13000 × *g* for 10 min, before removing 500 µL of sample for reverse-phase high-performance liquid chromatography analysis using a Zorbax^®^ SB-Aq column (250 × 4.6 mm, 5 µm; Agilent Technologies, USA) at a detection wavelength of 210 nm (Ni et al. 2013).

*In vivo*, the BNAS and SspB plasmids were co-transformed in *E. coli* BL21 (DE3). Recombinant strains were cultured in LB containing 100 μg/ml ampicillin and 50 μg/ml kanamycin at 37 °C. When the OD_600_ value reached 0.7, 0.1 mM IPTG was added and cells were incubated at 18°C for 20 h. The cells were collected by centrifugation and washed twice with PBS. To assess the catalytic efficiency of the BNASS, 10 mg/ml of cells was assayed for the ability to synthesize [mandelic](D:/Dict/6.3.69.8341/resultui/frame/javascript:void(0);) [acid](D:/Dict/6.3.69.8341/resultui/frame/javascript:void(0);) as described above. The standard assay mixtures contained 20 mM [mandelonitrile](D:/Dict/6.3.69.8341/resultui/frame/javascript:void(0);), 10 µM FMN, 100 mM PBS (pH 7.4), and 10 mg/ml of cells. After reaction at 30 °C with agitation at 200 rpm for 20 min under the blue light. 100 µL of 1 M HCl was added to stop the reaction, and centrifugation was performed at 13000 × *g* for 10 min, before removing 500 µL of sample for reverse-phase high-performance liquid chromatography analysis using a Zorbax^®^ SB-Aq column (250 × 4.6 mm, 5 µm; Agilent Technologies, USA) at a detection wavelength of 210 nm.
